# Transmural Ileal Fibroplasia Causing Mechanical Obstruction in a Dog: Surgical Management, Histopathology, and Molecular Findings

**DOI:** 10.3390/vetsci13020174

**Published:** 2026-02-09

**Authors:** Duhwan Park, Hyung-Seok Seo, Sangyul Lee, Kieun Bae, Young Jae Lee, Aryung Nam, Jung-Moon Kim, Hwi-Yool Kim

**Affiliations:** 1 Department of Veterinary Surgery, College of Veterinary Medicine, Konkuk University, Seoul 05029, Republic of Korea; ppdh888@konkuk.ac.kr (D.P.); gudtjrdldl@konkuk.ac.kr (H.-S.S.); tkddbf7918@konkuk.ac.kr (S.L.); kimjungm418@gmail.com (J.-M.K.); 2 Department of Veterinary Biochemistry, College of Veterinary Medicine, Konkuk University, Seoul 05029, Republic of Korea; kieun86@konkuk.ac.kr; 3 Department of Veterinary Internal Medicine, College of Veterinary Medicine, Konkuk University, Seoul 05029, Republic of Korea; hleeyj0726@gmail.com (Y.J.L.); aryung@konkuk.ac.kr (A.N.)

**Keywords:** small bowel obstruction, chronic enteropathy, transmural fibroplasia, hyperadrenocorticism, intestinal resection, *PDGFRB*

## Abstract

Small bowel obstruction (SBO) in dogs is most commonly caused by foreign bodies or neoplasia; however, SBO secondary to transmural fibroplasia remains a rare clinical complication of canine chronic enteropathy. This report describes the complex case of mechanical SBO caused by transmural ileal fibroplasia and inflammation at the ileocolic junction in a 9-year-old mixed-breed dog with concomitant hyperadrenocorticism (HAC). The primary presentation involved chronic severe weight loss and intermittent anorexia, contrasting with the acute presentation typical of most SBO cases. Imaging studies revealed severe, circumferential thickening up to 10 mm of the small intestine wall at the ileocolic junction (ICJ), resulting in complete luminal stricture and marked proximal dilation. Surgical intestinal resection and anastomosis were performed for alleviation of the obstruction, and histopathology confirmed severe mural and serosal enteritis with extensive fibroplasia extending into the adjacent mesentery, thereby excluding neoplastic processes. Quantitative PCR analysis demonstrated a significant upregulation of mRNA expression for *PDGFRB* and *FGFR* compared to normal tissue. Postoperative recovery was rapid; although soft feces persisted for one month, normal stool consistency was subsequently restored, and the patient achieved significant weight gain. This case underscores the diagnostic and therapeutic challenges associated with non-neoplastic, inflammation-driven SBO and suggests that activation of the PDGFR-β/FGFR pathways may play a key role in fibroplasia-related intestinal strictures, offering a novel molecular perspective beyond conventional SBO etiologies.

## Simple Summary

This case report describes a mechanical small bowel obstruction caused by transmural ileal fibroplasia at the ileocolic junction in a dog. Diagnostic imaging and histopathological examination confirmed a severe fibrotic stricture at the ileocolic junction, accompanied by marked dilation of the distal ileum, with no evidence of malignancy. Cancer Gene Expression analysis revealed a 2.38-fold increase in both *PDGFRB* and *FGFR* expression, suggesting a potential role for aberrant receptor tyrosine kinase signaling in the development of pathological fibroplasia. In addition, concurrent hyperadrenocorticism may have acted as a permissive systemic factor, contributing to the chronic fibrotic remodeling observed in this case.

## 1. Introduction

Small bowel obstruction (SBO) is a common and critical surgical condition in dogs, frequently resulting from mechanical causes such as foreign bodies, intussusception, or neoplasia [1,2]. This condition commonly presents with severe, acute clinical signs requiring immediate intervention. These clinical signs are often accompanied by typical radiographic findings of intestinal dilation and evident obstructive lesions on ultrasonography, occasionally showing loss of wall layering in cases of aggressive neoplasia or acute necrosis [3,4]. While chronic enteropathy (CE) is prevalent in canine practice [5,6], its progression to extensive transmural fibrosis and complete mechanical obstruction is uncommon, often requiring surgical intervention only in exceptional cases. When SBO does result from non-neoplastic inflammatory causes, it is commonly attributed to severe, end-stage enteritis or strictures resulting from chronic inflammation [1,2].

However, although cases of chronic intestinal pseudo-obstruction (CIPO) or functional obstruction secondary to chronic enteropathy have been reported in the veterinary literature [7,8,9]. To the authors’ knowledge, complete mechanical obstruction requiring surgical resection due to severe, non-neoplastic transmural fibroplasia with associated intestinal dilation remains exceptionally rare. The precise molecular mechanisms underlying such extensive fibroplasia in these uncommon cases remain poorly elucidated. In particular, the contribution of key profibrotic signaling pathways, including the PDGFR-β/FGFR pathways, to the development of canine intestinal strictures is largely unknown. Furthermore, the ileocolic junction (ICJ) is a particularly sensitive and anatomically complex region, and severe fibroplasia-induced SBO localized to this area poses a distinct diagnostic challenge [1,2].

Transmural fibroplasia in dogs refers to progressive fibrotic remodeling involving the full thickness of the intestinal wall; in chronic inflammatory conditions, this process may be associated with concurrent inflammatory infiltration of the submucosa, muscularis, and serosa, ultimately resulting in irreversible structural remodeling and luminal narrowing [10]. While similar fibroproliferative lesions, such as feline gastrointestinal eosinophilic sclerosing fibroplasia (FGESF) or its canine counterpart, idiopathic eosinophilic gastrointestinal mass, predominantly occur at the ileocolic junction (ICJ) [11,12], transmural fibroplasia driven by predominantly non-eosinophilic inflammatory infiltration remains incompletely characterized. Moreover, comprehensive clinicopathologic and molecular descriptions of such transmural fibroplastic processes resulting in true mechanical obstruction are scarce. Therefore, the purpose of this report is to describe a rare case of mechanical SBO caused by transmural ileal fibroplasia at the ICJ in a dog with chronic enteropathy and concomitant hyperadrenocorticism, and to provide molecular insight into the potential role of PDGFR-β and FGFR signaling pathways in the pathogenesis of non-neoplastic, inflammation-driven intestinal strictures.

## 2. Case Presentation

### 2.1. Clinical History and Physical Examination

The patient was a 9-year-old castrated male mixed-breed dog weighing 5.29 kg with a body condition score (BCS) of 3/9. The dog had been rescued from a shelter and lived in a multi-dog environment, which limited the owner’s ability to provide a comprehensive medical history or specific dietary details. The owner reported a long-standing history of severe, progressive weight loss and intermittent anorexia; however, no specific changes in fecal consistency were noted prior to presentation. Regular anthelmintic treatment had been maintained. Approximately two years before the current presentation, the patient had exhibited similar clinical signs and was diagnosed with chronic enteropathy. Although medical management was implemented, satisfactory long-term clinical control was not achieved. According to the owner, the weight loss had markedly worsened over the several weeks preceding the current presentation, prompting further evaluation. The dog had been newly diagnosed with hyperadrenocorticism (HAC) at a local veterinary clinic approximately one week prior to presentation, based on adrenocorticotropic hormone stimulation test results (pre-ACTH cortisol, 12.8 µg/dL; post-ACTH cortisol, 30.0 µg/dL; Vcheck cortisol assay), and trilostane therapy (2 mg/kg, PO, BID) was initiated.

On physical examination, the patient exhibited stable vital signs and a responsive mentation, with a mildly distended abdomen, thin skin, and generalized alopecia. A skin tent test and tacky mucous membranes were consistent with mild to moderate dehydration, estimated at approximately 6–7%. The only notable laboratory finding was a mild elevation in C-reactive protein (2.7 mg/dL; reference range: 0.0–1.0 mg/dL), as all other hematologic and serum biochemical parameters remained within normal limits. Direct fecal smear examination (both wet and dry mounts) was performed at the time of presentation and yielded no remarkable findings. To further exclude infectious causes of chronic enteropathy, a comprehensive fecal polymerase chain reaction (PCR) panel for common enteric pathogens (including bacteria, viruses, and protozoa) was conducted, and all results were negative. Serum cobalamin concentration was less than 150 ng/L (reference range: 284–836 ng/L), consistent with mild hypocobalaminemia. Intramuscular hydroxocobalamin (vitamin B12) was administered at diagnosis, followed by a weekly maintenance dose of 250 μg/kg. Although the owner reported severe and progressive weight loss, the precise duration and magnitude of this weight loss could not be reliably determined due to limited historical information. Advanced diagnostic imaging was therefore performed to assess the gastrointestinal tract and exclude an underlying malignancy.

### 2.2. Diagnostic Imaging

**Figure 1:**
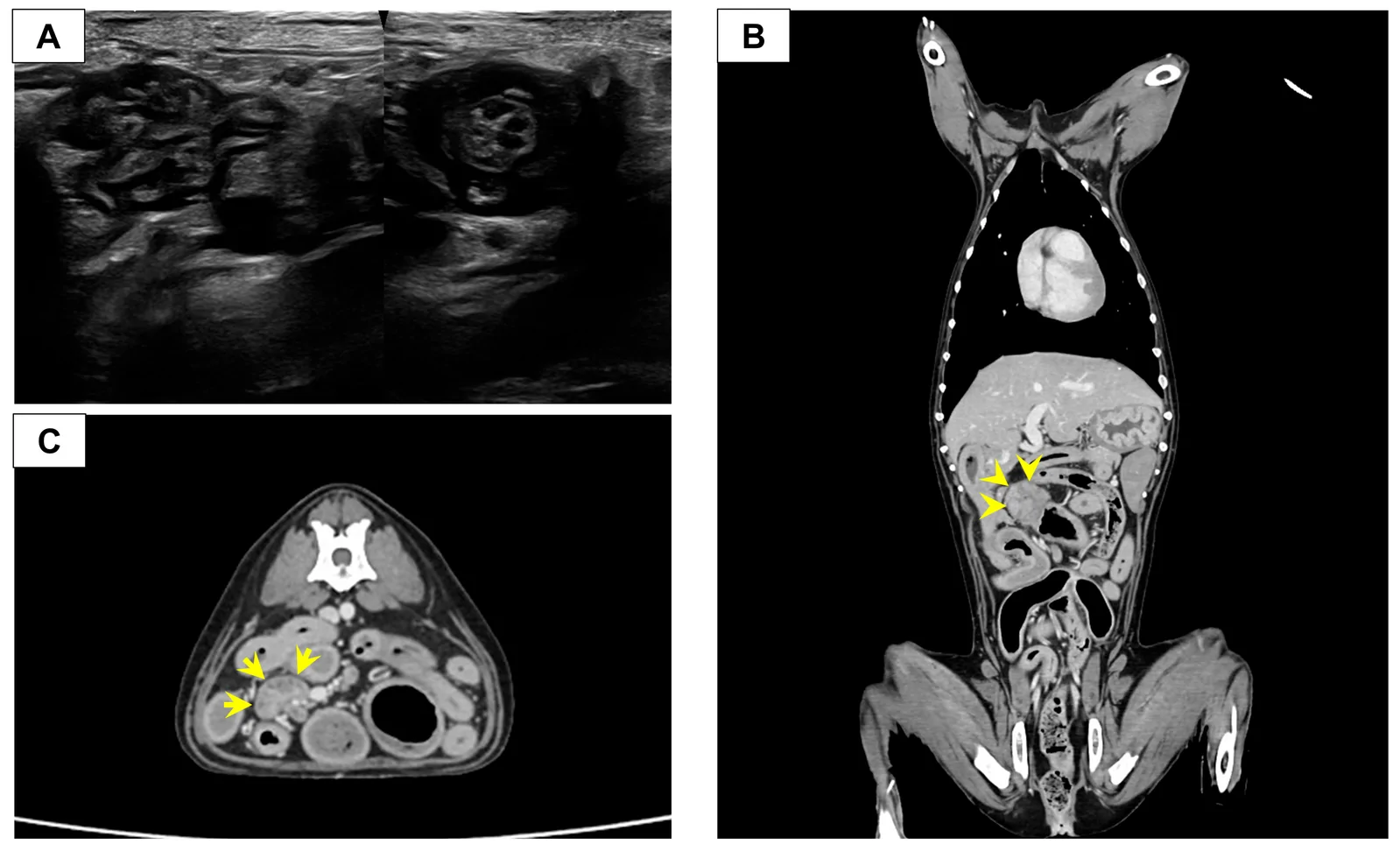
Diagnostic imaging (Computed Tomography (CT) and Ultrasonography) illustrating the ileocolic junction (ICJ) lesion and key associated diagnostic findings at the ICJ. (**A**) Sagittal (left) and Transverse (right) ultrasonographic image of the ICJ. (**B**) Coronal CT image (Delayed phase) of the ICJ. The image reveals severe, circumferential wall thickening (maximum 10 mm) resulting in non-discernible luminal patency (arrowhead). (**C**) Transverse CT image (Delayed phase) of the Jejunal lymph node (LN) adjacent to the lesion. The lymph node demonstrates significant enlargement (L × H × W = 20.9 × 12.5 × 9.5 mm), accompanied by thickening and heterogeneous contrast enhancement (arrow).

Abdominal radiographs obtained at presentation demonstrated segmental dilation of the small intestine, raising concern for a partial mechanical obstruction. Abdominal ultrasonography identified a localized lesion at the ileocolic junction (ICJ). Focal thickening of the ileal muscularis layer adjacent to the ICJ was noted, accompanied by an abnormal overlapping configuration (Figure 1A), raising suspicion for partial intussusception or an intestinal stricture. The intestinal wall layering was preserved, and no ultrasonographic findings were indicative of complete mechanical obstruction at the time of evaluation. Moderate enlargement of the regional lymph nodes was also noted, with heterogeneous hypoechoic parenchyma. The differential diagnoses considered included benign inflammatory disorders, such as chronic enteropathy or lipogranulomatous lymphangitis, as well as partial intussusception and intestinal neoplasia. Because the ultrasonographic findings were inconclusive and mechanical obstruction could not be ruled out, advanced diagnostic imaging was pursued for detailed characterization of the lesion and evaluation for possible malignancy.

Subsequent CT imaging confirmed a severe, circumferential, and symmetric wall thickening of the distal ileum (maximum thickness 10 mm, length approximately 23 mm). This severe pathology resulted in a non-discernible intestinal lumen at the lesion level, leading to the loss of luminal patency (Figure 1B). Proximal to the stricture, severe small intestinal dilation was confirmed (maximum diameter: 27.4 mm, small intestine (SI) to L5 vertebral body ratio = 3.3) with intraluminal high-attenuating material (small bowel feces sign), suggesting complete mechanical obstruction. Regional lymphadenopathy was also apparent; the jejunal lymph node adjacent to the ICJ lesion was significantly enlarged (20.9 × 12.5 × 9.5 mm) and exhibited heterogeneous contrast enhancement on CT (Figure 1C). Bilateral adrenal enlargement was also noted, measuring a maximum diameter of 7.6 mm at the cranial pole, which was consistent with the patient’s history of hyperadrenocorticism. Given the presence of complete mechanical obstruction and the persistent inability to exclude malignancy based on imaging findings, surgical intervention was required for both immediate alleviation and definitive histopathological diagnosis.

### 2.3. Surgical Intervention and Gross Findings

**Figure 2:**
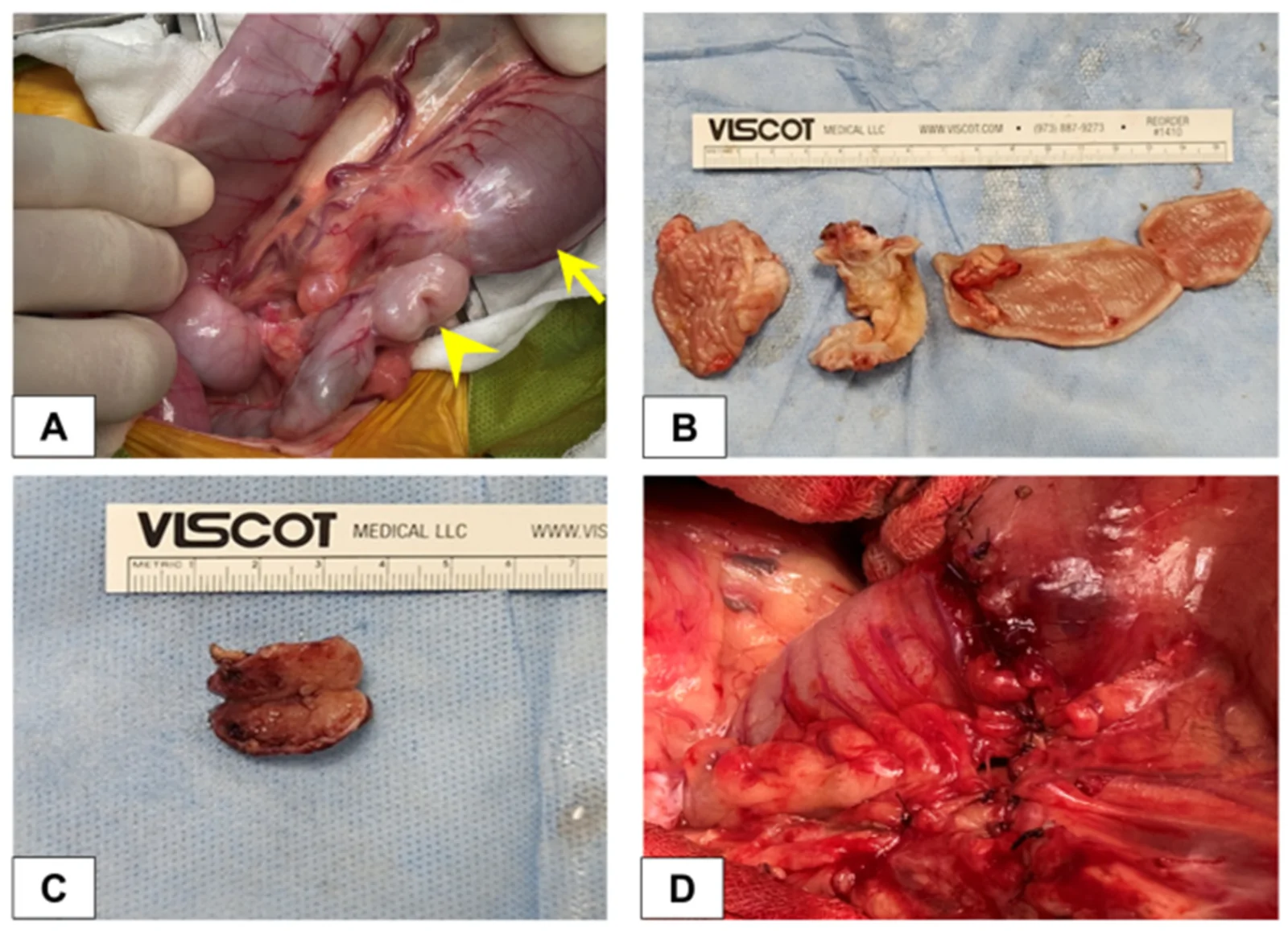
Intraoperative findings and gross pathology of the ileocolic junction (ICJ) lesion. (**A**) Intraoperative image demonstrating the severe lesion at the ICJ. The arrow indicates the markedly dilated distal ileum filled with stagnant intestinal contents, while the arrowhead indicates the cecum, confirming the lesion’s location at the ICJ. (**B**) Gross appearance of the resected intestinal segment, showing a markedly thickened and rigid distal ileal wall (left) compared with the normal-appearing colon (right). (**C**) Gross examination of the resected jejunal lymph node (LN) revealed generalized enlargement (approximately 22 mm). (**D**) Final appearance of the surgical site demonstrating the completed ileocolic anastomosis and closure of the mesentery.

Anesthesia was administered using a multimodal protocol. Premedication consisted of maropitant citrate (1 mg/kg, SC) and famotidine (1 mg/kg, IV). Prophylactic antibiotic coverage was provided with cefazolin sodium (22 mg/kg, IV) administered prior to the skin incision. For induction, a combination of midazolam (0.2 mg/kg, IV; Bukwang Pharm. Co., Ltd., Seoul, Republic of Korea) and propofol (4 mg/kg, IV; Hana Pharm Co., Ltd., Seoul, Republic of Korea) was utilized to facilitate endotracheal intubation. General anesthesia was maintained with inhaled isoflurane in 100% oxygen, while intraoperative pain management was provided via constant rate infusion of remifentanil (0.1–0.4 μg/kg/min, IV; Hana Pharm Co., Ltd., Seoul, Republic of Korea). Following a ventral midline celiotomy, surgical exploration revealed a markedly distended segment within the distal ileum, identified immediately proximal to the ICJ (Figure 2A). Intraoperatively, the affected segment of the distal ileum was found to be severely dilated, mimicking the diameter and appearance of the large intestine, and was entirely filled with stagnant intraluminal contents. Manual assessment of intestinal patency confirmed complete obstruction at the affected site. An ileocolic resection and anastomosis were performed with adequate margins include the entire area of fibroplastic thickening. The procedure included resection of the affected distal ileum, the ICJ, and the associated enlarged jejunal lymph nodes. A successful ileocolic anastomosis was confirmed following resection (Figure 2D). On gross examination, the lesion exhibited marked, circumferential wall thickening and firmness (Figure 2B). Gross enlargement of the regional jejunal lymph nodes was also confirmed, measuring approximately 22 mm in length (Figure 2C). Both lesional tissue and adjacent grossly normal intestinal tissue were separately collected and preserved for subsequent molecular analysis of Cancer Gene Expression (CGE).

### 2.4. Histopathology and Molecular Findings

**Figure 3:**
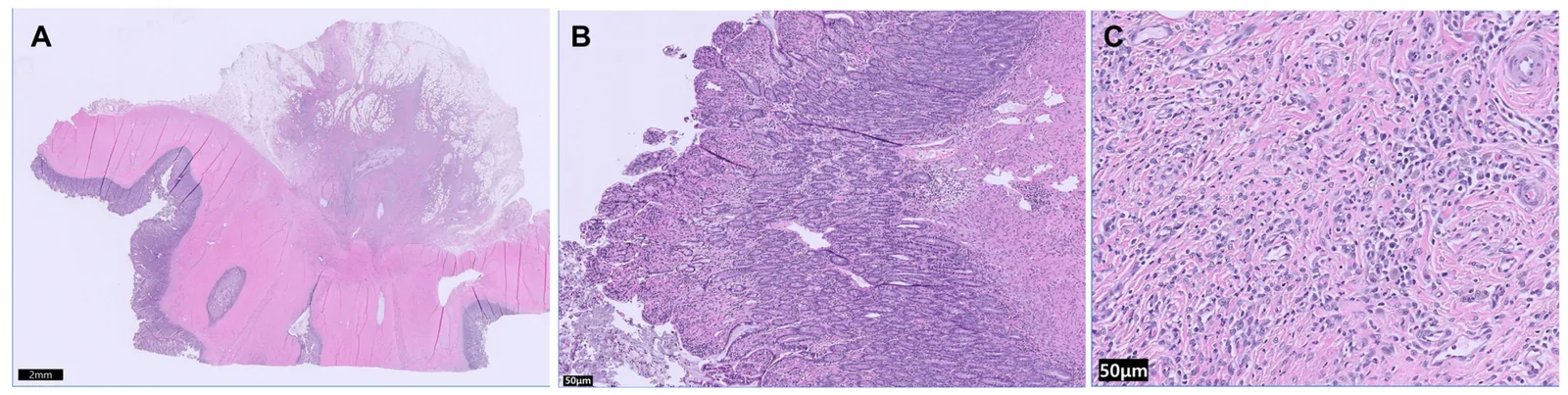
Histopathological and Molecular findings of the transmural ileal inflammation and fibrosis. (**A**) 5×, H&E, Full-thickness section demonstrating severe, transmural inflammation and fibroplasia extending into the mesentery (Scale bar = 2 mm). (**B**) 100×, H&E, Inflammation in the intestinal mucosa and submucosa (Scale bar = 50 μm). (**C**) 400×, H&E, Mesenteric inflammation and fibroplasia with dense collagen bundles and predominantly neutrophilic, plasmacytic, and histiocytic infiltrates (Scale bar = 100 μm).

Histopathological examination of the resected ileocolic segment and associated jejunal lymph nodes established the definitive diagnosis. Microscopic evaluation revealed severe, transmural inflammation and fibroplasia involving the ileal wall. The lesion was characterized by extensive fibroplasia affecting the mural and serosal layers, with extension into the adjacent mesentery (Figure 3A). The inflammatory component consisted predominantly of lymphoplasmacytic enteritis within the mucosa and submucosa, while the mesenteric fibroplastic regions contained a mixed chronic inflammatory cell population composed primarily of plasma cells and histiocytes (Figure 3B,C).

Histopathological analysis definitively ruled out malignancy, identifying the lesion as a non-neoplastic fibrotic intestinal stricture. The excised lymph nodes demonstrated reactive lymphoid hyperplasia, consistent with chronic, non-specific antigenic stimulation secondary to the adjacent enteritis. No histopathological evidence of metastasis or primary lymph node pathology was identified in the examined tissues.

**Figure 4:**
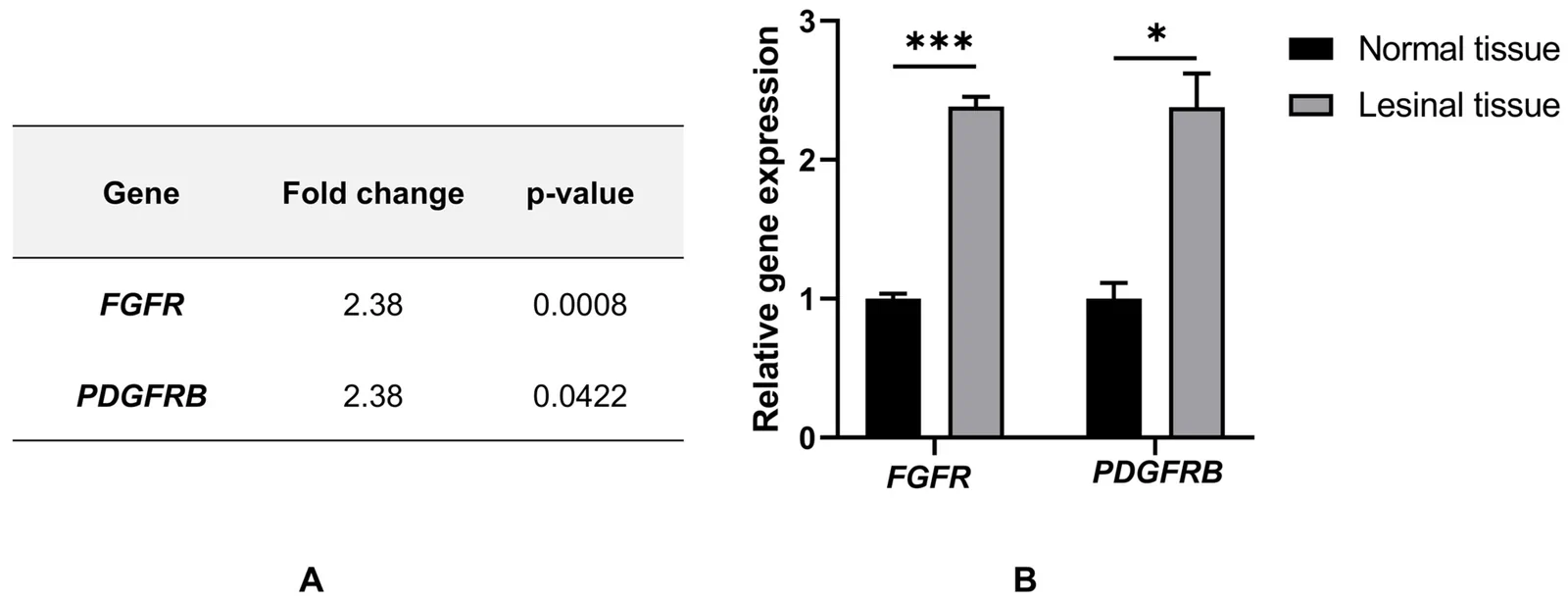
Relative expression of *FGFR* and *PDGFRB* in lesional tissue compared with normal tissue. (**A**) Summary of fold-change values and statistical significance (*p*-values) for *FGFR* and *PDGFRB* expression in lesional tissue relative to normal tissue. (**B**) Relative mRNA expression levels of *FGFR* and *PDGFRB*. Both genes exhibited a 2.38-fold increase in lesional tissue compared with normal tissue. Data are presented as mean ± SEM. Statistical significance was determined using Welch’s *t*-test, with *FGFR* showing *** (*p* < 0.001) and PDGFRB showing * (*p* < 0.05).

Given the extensive transmural fibroplasia observed on histopathologic examination, molecular analysis was subsequently performed to investigate the involvement of profibrotic receptor tyrosine kinase signaling pathways potentially contributing to this fibrotic response. Cancer Gene Expression (CGE) analysis was conducted by first extracting total RNA using TRIzol reagent, followed by DNase treatment and cDNA synthesis. Gene expression was then quantified using the VDx^®^ Companion Animal TRK qRT-PCR Kit (Cat. No. NC-CTG, Median Diagnostics, Chuncheon, Republic of Korea) according to the manufacturer’s instructions. Expression levels were normalized to GAPDH (Cy5), and fold changes were calculated by setting the expression of normal tissue to 1. Differences between normal and tumor tissues were assessed using Welch’s *t*-test based on ΔCt values, with *p* < 0.05 considered statistically significant. Quantitative analysis demonstrated significant upregulation of mRNA expression for both fibroblast growth factor receptor (*FGFR*) and platelet-derived growth factor receptor beta (*PDGFRB*) in fibrotic lesional tissue compared with adjacent normal tissue (Figure 4A). Statistical evaluation of the bar graphs confirmed a significant increase in *PDGFRB* expression (*p* < 0.05), while *FGFR* showed a highly significant elevation (*p* < 0.001) (Figure 4B). Interestingly, the mRNA expression levels of both genes were increased by approximately 2.38-fold in the lesion.

### 2.5. Postoperative Course and Outcome

The patient recovered from the surgical procedure without major perioperative complications. However, on postoperative day 1, the patient developed hypoalbuminemia (albumin, 1.6 g/dL; reference range, 2.3–4.0 g/dL), necessitating administration of fresh frozen plasma (FFP) at a dose of 10 mL/kg over a 6 h period to support plasma oncotic pressure and postoperative recovery. Following FFP administration, serum albumin concentration increased to 2.1 g/dL. Oral feeding was initiated 12 h postoperatively using a low-fat gastrointestinal liquid diet, divided into three to four meals per day, targeting 1.2× resting energy requirement (RER). The patient exhibited normal appetite and activity levels. Postoperative antimicrobial therapy consisted of cefoxitin (30 mg/kg, IV, TID), metronidazole (15 mg/kg, IV, BID), and enrofloxacin (20 mg/kg, IV, SID) for the first 3 days, followed by oral administration of cephalexin (22 mg/kg, PO, BID), metronidazole (15 mg/kg, PO, BID), and enrofloxacin (20 mg/kg, PO, SID) for an additional 9 days. Postoperative analgesia was maintained with remifentanil (0.1–0.4 µg/kg/min, CRI) for 48 h. A transient period of soft, unformed feces persisted for approximately one month postoperatively, which was successfully managed with dietary and symptomatic therapy, including supplementation with dietary fiber (psyllium) and an intestinal adsorbent (smectite). Following resolution of the gastrointestinal signs, the patient demonstrated gradual but sustained improvement in body condition. At the 1-month postoperative evaluation, body weight had already increased to 7.69 kg (BCS 5/9). Concurrent management of hyperadrenocorticism (HAC) was controlled throughout the postoperative period with trilostane. The initial dosage was 2 mg/kg, administered orally twice daily, and was adjusted to 1.5 mg/kg twice daily following adrenocorticotropic hormone stimulation testing at 1 month postoperatively. At the 8-month postoperative follow-up, the patient remained clinically stable, and no recurrence of gastrointestinal signs was observed. Follow-up imaging demonstrated an intact anastomosis with preserved normal intestinal wall layering, without evidence of stenosis or abnormal wall thickening. The long-term prognosis was favorable, with complete resolution of the mechanical intestinal obstruction and sustained clinical improvement.

## 3. Discussion

Small bowel obstruction (SBO) typically allows for a straightforward diagnosis through its acute clinical manifestations and distinct imaging features [1,2]. In cases of acute mechanical obstruction, the differential diagnosis primarily includes intraluminal foreign bodies, intussusception, and various neoplastic masses [1,2]. Inflammatory and fibroproliferative masses, such as idiopathic eosinophilic gastrointestinal masses or granulomatous masses, can mimic malignancy by forming obstructive lesions [12,13]. Among these, the idiopathic eosinophilic gastrointestinal mass—which frequently occurs at the ileocolic junction (ICJ)—is a particularly important differential diagnosis given its pathophysiological and anatomical similarity to the present case [12]. It is typically characterized by a heavy infiltration of inflammatory cells, particularly eosinophils, across all layers of the intestinal wall, accompanied by widespread fibroplasia [12]. In the present case, several atypical features contributed to diagnostic uncertainty; specifically, the mechanical obstruction at the ileocolic junction (ICJ) was driven by extensive fibroplasia associated with a non-eosinophilic inflammatory infiltrate, rather than the more commonly reported eosinophilic or neoplastic lesions. Furthermore, although intraluminal foreign bodies are a common cause of such obstructions, no history suggestive of foreign body ingestion, prior gastrointestinal surgery, or pica-like behavior was identified in this patient. Ultimately, the definitive diagnosis of transmural ileal fibroplasia was established through the integrated assessment of preoperative imaging and histopathology, confirming severe circumferential thickening and transmural fibroplasia without evidence of malignancy.

Abdominal ultrasonography demonstrated preserved intestinal wall layering—a characteristic that initially lowered the suspicion of high-grade malignancy [14]. Instead of a discrete foreign body or a well-defined mass, the obstructive lesion manifested as a localized, inflammatory wall thickening at the ICJ. The anatomical complexity of the ileocolic junction predisposes this region to severe inflammatory conditions as well as neoplastic processes, including lymphoma, gastrointestinal stromal tumors (GISTs), and adenocarcinoma, thereby complicating the diagnostic process in the present case [1,15]. Although the exact degree of mechanical obstruction was difficult to assess by initial ultrasound, CT confirmed a complete luminal obstruction at the ICJ with distal ileal dilation. Furthermore, a small intestine (SI) to L5 vertebral body ratio (SI/L5) of 3.3 provided an objective, quantitative measure of the severity of small bowel dilation/thickening, supporting the clinical suspicion of severe bowel obstruction [3,4,16]. While this metric reflects luminal distension instead of mural pathology, it functioned as a supplementary indicator to assess obstruction severity. CT also revealed enlarged jejunal lymph nodes (approximately 20 mm) with heterogeneous contrast enhancement. Combined with the severe obstructive findings, these lymph node characteristics maintained the suspicion of malignancy until surgical exploration. Consequently, surgical intervention consisting of resection and anastomosis of the affected bowel segment, including the ICJ, along with regional lymphadenectomy, was indicated not only for therapeutic resolution of the obstruction but also to obtain a definitive histopathological diagnosis necessary to differentiate inflammatory stricture from intestinal neoplasia.

Histopathological examination of the excised ileocolic junction (ICJ) segment and regional lymph nodes definitively excluded malignancy, confirming the diagnosis of transmural enteritis with extensive fibroplasia. The predominant pathological feature was marked fibroplasia involving the intestinal wall, extending into the adjacent mesentery. Notably, unlike an idiopathic eosinophilic gastrointestinal mass, there was no significant or predominant infiltration of eosinophils observed within the lesions. Lymph node histopathology demonstrated only reactive lymphoid hyperplasia consistent with chronic, non-specific inflammatory stimulation. Chronic enteropathy (CE) typically correlates with functional obstruction, such as chronic intestinal pseudo-obstruction (CIPO) induced by underlying enteritis [7,8,9]. Progression to severe, localized mechanical obstruction via fibrotic stricture remains rarely documented. Although inflammatory mechanical obstruction has been described in association with granulomatous enteritis [13], the luminal occlusion in this patient resulted from extensive fibroplasia and chronic inflammation instead of a granulomatous process. Given the severity of this fibrotic response, further molecular investigation was warranted to identify the underlying drivers of such extensive tissue remodeling.

Intraoperatively, the distal ileum was markedly distended with static luminal contents, and direct palpation confirmed complete physical occlusion at the ICJ. The presence of static intraluminal contents correlated with the small bowel feces sign identified on preoperative CT imaging, suggesting that chronic functional ileus may have coexisted, secondarily induced by the severe underlying mechanical stricture pathology at the ICJ [17,18]. Postoperatively, the patient exhibited a transient period of hypoproteinemia. In the context of chronic enteropathy, this was interpreted as a multifactorial consequence of severe chronic inflammation, postoperative changes, and potentially transient protein malabsorption during the initial recovery phase [1,19]. To address the acute protein deficit and provide oncotic support, a fresh frozen plasma (FFP) transfusion was administered in the early postoperative period. The condition was successfully managed with this supportive care and resolved as the patient’s clinical status stabilized.

Cancer Gene Expression (CGE) analysis was conducted to investigate the molecular basis of fibroplasia and to assess potential oncogenic drivers that could not be ruled out preoperatively. Compared with adjacent normal intestinal tissue, the affected segment demonstrated significantly increased expression of *PDGFRB* and *FGFR*. These molecular findings support a pathogenic link between aberrant receptor tyrosine kinase signaling and the marked fibroplastic response responsible for the mechanical obstruction observed in this case. These results, showing a 2.38-fold increase in both *PDGFRB* and *FGFR* expression, support the conclusion that receptor tyrosine kinase signaling was a key driver of the abnormal fibroplasia observed in this case. PDGFR-β is a receptor tyrosine kinase (RTK) that plays a central role in the proliferation and migration of mesenchymal cells, such as fibroblasts [19,20,21]. While its high expression is often associated with malignancy [22,23], in non-neoplastic contexts, it serves as a key mediator transitioning chronic inflammation into pathological fibrosis. The concurrent upregulation of FGFR likely further accelerated neovascularization and the formation of the rigid fibrotic stricture [19,20,21]. Although confirmation of protein expression via immunohistochemistry (IHC) was limited by antibody availability, the marked mRNA upregulation detected by qPCR suggests that these molecular pathways likely played a significant role in the pathogenesis of the fibrotic stricture in this case.

The patient’s concurrent endocrine disease, Hyperadrenocorticism (HAC), necessitates discussion as a systemic factor potentially contributing to the severity of the fibrotic process. Although HAC is classically associated with anti-inflammatory and anti-fibrotic effects of glucocorticoids, increasing evidence suggests that chronic glucocorticoid excess may paradoxically contribute to pathological fibrosis by impairing normal tissue repair mechanisms [24,25]. Within the gastrointestinal tract, excessive glucocorticoid exposure may suppress the acute inflammatory signals essential for mucosal repair, thereby diverting the healing process toward a maladaptive, fibroproliferative state [19,24]. In the present case, the concurrent hyperadrenocorticism (HAC) may therefore have acted as a permissive systemic factor that exacerbated chronic intestinal injury and promoted pathological fibroplasia, rather than serving as a direct profibrotic driver. In this context, the severely obstructive fibrotic stricture observed at the ileocolic junction was likely not solely the result of localized PDGFR-β–driven stromal activation but may have been amplified by a systemic endocrine environment that impaired normal tissue healing and favored chronic fibrotic remodeling. Importantly, the interaction between glucocorticoid-mediated alterations in tissue repair and local profibrotic signaling pathways, including PDGFR-β and FGFR activation, remains speculative and cannot be definitively established from a single case.

Accordingly, several limitations should be acknowledged. Although increased *PDGFRB* and *FGFR* expression may provide a molecular context, their precise contribution to ileocolic strictures remains difficult to generalize from a single patient. Furthermore, functional validation of these molecular findings, such as protein-level expression analysis or IHC, was not performed, which limits the interpretation of the biological significance of the observed mRNA upregulation. Consequently, future studies involving larger cohorts and protein-level validation, including immunohistochemistry, are warranted to clarify the distribution and functional relevance of these receptors and to determine whether such molecular signatures are consistently observed in comparable inflammatory lesions. Additionally, while modulation of receptor tyrosine kinase–associated pathways may represent a potential area of future investigation, the clinical relevance of such approaches cannot be inferred from the present data. Additional experimental and clinical studies will be necessary to clarify the role of these molecular pathways in the pathogenesis of fibrotic intestinal obstruction associated with chronic inflammatory disease. Importantly, this case underscores that severe fibroplasia-driven ileocolic obstruction can occur in the absence of eosinophilic or neoplastic infiltration, highlighting the need to consider non-neoplastic, fibroproliferative etiologies in the differential diagnosis of small bowel obstruction in dogs.

## 4. Conclusions

In conclusion, this case describes a rare incidence of complete mechanical small bowel obstruction caused by a severe, fibrotic stricture at the ileocolic junction. The patient’s atypical presentation, characterized by non-specific, chronic signs, posed a significant diagnostic challenge where delayed intervention could have rapidly led to a life-threatening state. Molecular analysis revealed that this severe fibroplasia was driven by the overexpression of the PDGFR-β/FGFR pathway, a finding suggestive of a pathological tissue remodeling process potentially exacerbated by the patient’s concurrent HAC. This case highlights that even in the presence of non-specific clinical signs, definitive surgical resection and histopathological confirmation are mandatory for the immediate therapeutic resolution of small bowel obstruction and for differentiating this inflammatory disease from malignancy.

## Data Availability

The original contributions presented in this study are included in the article. Further inquiries can be directed to the corresponding author.

## Abbreviations

The following abbreviations are used in this manuscript:

SBO	Small bowel obstruction
ICJ	Ileocolic junction
HAC	Hyperadrenocorticism
PDGFR-β	Platelet-derived growth factor receptor- β
FGFR	Fibroblast Growth Factor Receptor
CE	Chronic enteropathy
